# Binge eating attitudes in community adolescent sample and relationships with interview-assessed attachment representations in girls: a multi-center study from North Italy

**DOI:** 10.1007/s40519-021-01183-8

**Published:** 2021-04-12

**Authors:** Cecilia Serena Pace, Stefania Muzi, Laura Parolin, Alberto Milesi, Giacomo Tognasso, Alessandra Santona

**Affiliations:** 1 Department of Educational Sciences (DISFOR), University of Genoa, Corso Andrea Podestà, 2, 16128 Genova, GE Italy; 2 Department of Psychology, University of Milano-Bicocca, Piazza dell’Ateneo Nuovo, 1, Milan, Italy

**Keywords:** Binge eating, Attachment, Adolescence, Friends & Family Interview, Community sample

## Abstract

**Purpose:**

To compare community girls at risk and not at risk for binge eating (BE) in attachment representations through a narrative interview and to test the predictive role of attachment pattern(s) on the risk of binge eating among community girls.

**Methods:**

From 772 community adolescents of both sexes (33% boys) screened through the Binge Eating Scale (BES), 112 girls between 14 and 18 years, 56 placed in a group at risk for binge eating (BEG), and 56 matched peers, not at risk (NBEG), were assessed in attachment representations through the Friends and Family Interview (FFI).

**Results:**

(1) Compared to NBEG, girls in the BEG showed more insecure-preoccupied classifications and scores, together with lower narrative coherence, mother’s representation as a secure base/safe haven, reflective functioning, adaptive response, and more anger toward mother. (2) Both insecure-dismissing and preoccupied patterns predicted 15% more binge-eating symptoms in the whole sample of community girls.

**Conclusions:**

Insecure attachment representations are confirmed risk factors for more binge eating, affecting emotional regulation and leading to “emotional eating”, thus a dimensional assessment of attachment could be helpful for prevention and intervention. Implications and limits are discussed.

**Level of evidence:**

III. Evidence obtained from cohort or case–control analytic studies

## Introduction

Since its addition to the last edition of the Diagnostic and Statistical Manual of Mental Disorder (DSM-5), research on Binge Eating Disorder (BED) constantly increased [1]. Its definition entails brief, recurrent, and psychologically stressful overeating episodes, alongside the perception of a loss of control on food intake, but without consequent compensatory behaviors like in bulimia [1, 2].

Studies revealed that BED is the highest common eating disorder (ED), with a lifetime prevalence of 1–3%, compared to 0.2–1.4% of anorexia nervosa (AN) and 0.6–1.9% of bulimia nervosa (BN) [2–4], prevailing among female gender like other EDs. Males seem underrepresented in the literature concerning ED [5], which seems to be linked to different issues in the disorders’ conceptualization and clinical practice. Indeed, until the Fourth edition of the DSM, EDs criteria were strongly “female-centric” [6, 7], thus research was more focused on female samples [8]. According to Støving and colleagues [9], clinicians are less aware of EDs clinical manifestations in males, and they tend to undervalue its diagnosis. Moreover, few men are likely to seek help for their eating behaviors. However, since the fifth edition of DSM [1], there has been a reported 28.9% increase in EDs diagnoses in men during the lifespan. These observations highlight the difficulties in collecting data about EDs in men and underline the importance of providing meaningful data.

Generally, BED is associated with highly negative physical and psychological disease, as obesity, diabetes, chronic pain, depression, anxiety, bipolar disorders and substance abuse, leading to severe social impairment and lower quality of life [1, 4, 10, 11]. Perspective and longitudinal studies [12, 13] highlighted that BED in adulthood is strongly associated to binge-eating (BE) behaviors and externalizing symptoms in adolescence. Therefore, exploring BE attitudes in community adolescent samples may be highly relevant to think and realize preventive interventions, and to examine psychological vulnerabilities that could be connected to BE.

One of the psychological factors that was mostly associated with all EDs, is insecure attachment [14, 15]. The attachment theory [16] postulates that a child develops an early attachment bond with a specific caregiver, internalized as an internal representation of the self, other, and their mutual relationship (IWM; Internal Working Model). Knowingly or not, IWMs will guide expectations, feelings, and behaviors in other significant relationships until adulthood [16]. Moreover, throughout life, IWMs grant access to information related to the attachment system, in terms of memories, thoughts, and reflections. Their influence can be captured by interviews like the Adult Attachment Interview (AAI) [17] that highlight interviewees' discourse style when speaking about attachment experiences and relationships. Specifically, if caregivers pay attention and coherently respond when the child’s express his/her attachment needs, the child would form a secure IWM, meaning an experience of the self as worthy of love and attention, the caregiver as available and responsive, the significant relationship as a source of gratification and help [16]. Secure IWM can be seen in adults able to value and invest in attachment relationships in a good balance with self-realization and to remember and speak about their early attachment relationships in an open, balanced, detailed, and carefree way [17, 18]. On the contrary, unfavorable early parent–child relationships predispose to develop insecure IWMs. When the child minimizes the attachment need of proximity in favor of exploration to avoid the caregiver’s rejection, an avoidant IWM is more probable. Avoidant IWMs may develop in dismissing adults more prone to devalue, idealize or derogate attachment relationships and experiences in favor of self-strength, and speak about them in an untruthful, generic, and too concise way [17, 18]. Else, when the child shows hyperactivation and vigilance to attachment-related stimuli, thus inhibiting exploration from seeking continuous proximity with an unpredictable and discontinuous caregiver, an ambivalent/resistant IWM is more probable. Ambivalent/resistant IWMs might foster entangled/preoccupied adults that limit their independence and excessively emphasize unsatisfying attachment relationships and talk about them in angry, vague, and confusing ways [18]. Finally, when the child experiences attachment traumas as parental loss, abandonment, neglecting, abusive and frightened/frightening caregivers, he/she may fail to organize a unique, coherent attachment strategy, thus developing a disorganized IWM. An early disorganized IWM might harbor disorganized (i.e., unresolved and fearful) adults that live long-life attachment relationships in a fearful way and breakdown when attachment traumatizing experience (i.e., loss, abuse) were recalled [18].

Moreover, IWMs are responsible for individuals’ emotion regulation and distress perception in the attachment context [19]. Indeed, individuals with secure attachment can regulate their emotions also thanks to secure IWM that permits them to have a good representation of the self, the others, and the relationships which are also crucial dimension in the development of personality disorders [20]. Consequentially, individuals with avoidant/dismissing attachment patterns will probably use avoidant emotion regulation strategies, and an individual characterized by ambivalent/preoccupied attachment patterns will be primarily incapable of modulating his emotional state [21]. In this way, the attachment can be used as a theoretical framework for comprehending BE, meaning that maladaptive IWM coming from insecure attachment is responsible for interpersonal problems that often result in negative affect. Negative affect is not processed maturely and adaptively by individuals with insecure attachment styles. Some individuals then tend to regulate this negative emotional state with food, which often results in loss of eating control, i.e., BE [22]. Using the attachment theory to understand BE is consistent with an interpersonal perspective: secure relationships are essential human needs fundamental to psychological well-being. Indeed, a conceptual framework on EDs based on the attachment theory would suggest that negative social evaluation trigger eating disturbances [23].

In the last 30 years, more than 70 studies, several reviews, and meta-analyses highlighted more attachment insecurity and disorganization both in clinical adults and adolescents with EDs, and in community samples with higher eating disordered behaviors [14, 15, 24–31]. However, few studies focus specifically on BED, probably due to its recent recognition as a separate diagnosis [1].

Studies on clinical adults with BED suggest a significant presence of disorganized categories in their AAIs [32], as well as better intervention outcomes, were related to reductions of patients’ attachment insecurity, both in the form of anxiety or avoidance [33], while no attachment studies on clinical adolescents with BED were found [34–36]. Studies on non-clinical samples showed relations between BE behaviors, both in adults and adolescent, and higher attachment insecurity, in terms of anxiety, preoccupation, avoidance and fearfulness [14] measured through self-report questionnaires [37–39], which revealed biases such as social desirability, situational issues, and casual responses. Only a recent study on community adolescents assessed IWMs through an interview, highlighting greater attachment preoccupation in at risk of BE girls compared to their not at risk of BE peers, as well as an independent predictive role of both preoccupied and dismissing attachment patterns on higher presence of BE behaviors [40].

Given that adolescence is a critical stage where psychopathology might develop and BED might arise [3, 41, 42], this contribution aims at expanding the current knowledge on the associations between attachment and BE attitudes in a non-clinical adolescent sample. Moreover, given that almost all studies with non-clinical groups employed self-report questionnaires [36], we used an age-adapted interview exploring attachment styles to overcome the biases mentioned above and surpass the superficial focus on attachment-only classifications. Indeed, we considered a microanalysis of attachment narratives in crucial dimensions, such as reflective functioning [35], peer relationships [32], and affective/emotion regulation [25, 38].

More specifically, we hypothesized that: (1) participants resulting at moderate/high risk of BE would show higher insecurity, especially in terms of higher preoccupied patterns and related scales, than peers being non at risk of BE. Moreover, at the explorative level, we examined whether BE groups would show lower scores in specific attachment-related dimensions, such as reflective functioning, self-esteem, peer relationships, and affective/emotion regulation; (2) both preoccupied and dismissing attachment patterns would be predictive of more BE behaviors.

## Methods

### Research design and participants

The research design of the current study included two waves of data collection. In the first wave, 772 community adolescents (33% males, aged 13–19 years, mean [*M*] = 15.6, standard deviation [SD] = 1.2) from high schools in Northern Italy, respectively 385 in the urban areas of Liguria (38% males, *M*_age_ = 15.59, SD_age_ = 1.1) and 387 in the urban areas of Lombardy (28% males, *M*_age_ = 15.7, SD_age_ = 1.3) were assessed on BE behaviors via the Binge Eating Scale (BES). No difference on BES scores neither on demographic variables was found between participants from Liguria and Lombardia.

Based on this screening of the first wave, 56 girls and one boy were selected because their BES total score overpassed the cut-off score of 17, index of a moderate risk of binge eating in adolescents [43, 44]. Given that the only boy at risk refused to participate, the second wave, 15 days later, included only 112 girls: 56 female adolescents (7.3%) between 14 and 18 years were considered part of the BE group (BEG) and matched for age and gender with 56 peers without BE (non-binge eating group = NBEG).

### Variables and measures

An ad hoc social-demographic questionnaire was used to collect demographic information about participants.

To evaluate the risk of BE in community adolescent sample, the Italian version of the BES [43, 44] was used. The BES is a well-known Likert-type self-report to measure specifically the presence of binge-eating behaviors and features indicative of an eating disorder, and it includes 16 items based on behavioral characteristics (e.g., amount of food consumed) and emotional and cognitive features. Scores range from 0 to 46: a score of 17 is a cut-off for the presence of a BED. The Italian version of the BES has been validated and has good internal consistency reliability (Cronbach’s alpha 0.89) with a moderate mean inter-item correlation (*r* = 0.34) [44].

To assess attachment representations, we administered the Friends and Family Interview (FFI) [45, 46]. The FFI is a semi-structured interview asking adolescents (aged 11–17) a set of 27 questions about themselves and their relationships with the most significant individuals in their lives, including parents, best friends, siblings and favorite teacher. FFIs were videotaped and transcribed verbatim and transcripts were rated by expert and reliable coders (first and second authors) according to the FFI coding system [15].

Based on the highest score, it is possible to highlight one of the four attachment classifications: secure, insecure-dismissing, insecure-preoccupied, insecure-disorganized. The FFI coding system also includes the following dimensional scales: (1) coherence, based on Grice’s maxims of good conversation, such as truth, economy, relation, and manner, plus overall coherence; (2) reflective functioning, in terms of developmental perspective, theory of mind, and diversity of feelings; (3) evidence of secure base (father, mother, and other significant figure); (4) evidence of self–esteem, in terms of social competence, school competence, and self-regard; (5) peer relations, intended as frequency of contact and quality of best friendship; (6) sibling relations, in terms of warmth, hostility, and rivalry; (7) affective regulation in attachment relationships, intended as idealization, role reversal, anger, derogation, and adaptive response; and (8) differentiation of parental representations. Every scale and classification was scored on a 7-point scale from 1 to 4 including midpoints (1 = no evidence; 2 = mild evidence; 3 = moderate evidence; 4 = marked evidence). The FFI showed good psychometric properties in terms of inter-rater reliability, factor structure, between country invariance, content, discriminant and convergent validity [46]. For this study, two reliable coders evaluated 25% of the interviews (*n* = 28) and obtained a Cohen’s *k* = 1 (*p* < 0.001) on the four‐way classification system. Only one coder evaluated the remaining FFIs.

### Procedure

The study procedure was approved both by the Research Ethical Committee of the Department of Educational Sciences, University of Genoa and by the Ethical Committee of Milano-Bicocca University, in line with the Declaration of Helsinki. All families and participants were informed of the university staff's research goals. In line with ethical requirements, it was emphasized that participants' cooperation was voluntary, without providing compensation, and that their answers were confidential and used only for the study. Participants, recruited through schools and voluntarily agreed to participate in the study, were requested to return to the research team a written informed consent signed by their parents before assessments were administered. In the 1st wave, adolescents completed questionnaires collectively during school hours, while in the 2nd wave, participants were interviewed in an individual session with trained Master Psychology students at school.

### Data analyses

Statistical analyses were conducted using the Statistical Package for Social Science (SPSS; Version 21.0) software. Chi-square test was used to compare BEG e NBEG on categorical variables, such as FFI’s attachment classifications, Cramer’s Phi (*φc*) as a measure of effect size for 2 × 2 comparisons, and Cramer’s *V* for more than 2 categories. *t*-Test was used to perform comparison on continuous variables’ scores, such as FFI’s dimensional scales. Second, to explore the correlation between BES and FFI attachment patterns’ scales, Spearman’s correlations were computed on the whole group (*N* = 112). Based on them, a multiple regression analysis was performed to evaluate the predictive role of attachment patterns on BES score, reporting Cohen's *f*^2^ as a measure of effect size (*f*^2^ ≥ 0.02 small effect, ≥ 0.15 medium, and ≥ 0.3 large).

## Results

### First wave

Overall, 57 (7.4%) community adolescents (*n* = 23, 96% females from Liguria, and *n* = 34 from Lombardia, all girls) resulted being at risk of BED (i.e., BES scores > 17). More specifically, 1.7% (*n* = 13) reported severe symptoms and 5.7% (*n* = 44) reported moderate symptoms. No differences on BES scores neither on demographic variables were found between participants from Liguria and Lombardy. Table 1 shows demographics in BEG (*n* = 56, girls as the only one boy refused to take part further) and NBEG (*n* = 56, girls): no group differences in matching variables, except for BES scores, were found.

**Table 1:** Matching characteristics of 112 community girls divided in two groups as at risk for binge eating (BEG) and not at risk (NBEG)

	BEG		NBEG		*t*_(df)_	*p*		95% CI	
Matching variables	*M*	SD	*M*	SD				LL	UL
BES/total score	23.30	4.90	5.50	4.30	20.10_(106)_	0.000		16.1	19.6
Age	16.4	1.3	16.4	1.3	0.07_(109)_	0.900		− 0.47	0.51
Siblings (number)	1.10	.82	1.10	0.72	0.12 _(108)_	0.900		− 0.27	0.31

	*n*	%	*n*	%	*χ*^2^_(df)_	*p*	Cramer’s *V*	LL	UL
*Body mass index*									
Underweight	9	16	10	18	2.57_(2)_	0.276	0.12	0.28	0.30
Normal	33	59	36	64					
Overweight	6	11	3	5					
No response	8	14	7	12					
*Parents*									
Cohabiting	38	68	44	78	18.20_(1)_	0.181	0.10	0.20	18.2
Separated	14	25	10	18					
No response	4	7	2	3.5					

*n* = 56 in each group. At risk with scores for binge eating > 17 in the Binge Eating Scale (BES); *χ*^2^ test was performed on percentage distribution with values > 5

### Second wave

As Table 2 shows, BEG obtained significantly more insecure classifications (52%) than NBEG (25%) in the two-way system and three-way system, and it was the only group showing one disorganized classification.

**Table 2:** Comparison on distribution of attachment categories by the Friends & Family Interview in 112 community girls at risk for binge eating (BEG) and not at risk (NBEG)

	BEG		NBEG		*χ*^2^_(df)_	*p*	Cramer’s *V*	95% CI	
	*n*	%	*n*	%				LL	UL
*2-Way*					15.39_(1)_	0.000	0.28	1.7	0
Secure	27	48	42	75					
Insecure	29	52	14	25					
*3-Way*					14.69_(2)_	0.001	0.27	0.000	0.002
Secure-autonomous	27	48	42	75					
Insecure-dismissing	12	21	8	14					
Insecure-preoccupied	16	28	6	11					
Disorganized	1	1.7	0	0					

*n* = 56 in each group. *χ*^2^ test was performed on percentage distribution with values > 5, thus disorganized categories were not compared

The comparison on the FFI dimensional scales reported in Table 3 highlighted that BEG obtained higher scores than NBEG in insecure-preoccupied patterns and anger towards the mother, and lower scores on scales of coherence in terms of relation, reflective functioning in terms of diversity of feelings toward father, mother as a secure base/safe haven, self-regard and adaptive response.

**Table 3:** Comparison of scores on the FFI’s scales of 112 community girls divided in two groups as at risk for binge eating (BEG) and not at risk (NBEG)

	BEG		NBEG		*t*_(df)_	*p*	95% CI	
	*M*	SD	*M*	SD			LL	UL
*Patterns*								
Secure-autonomous	3.09	4.44	2.99	0.75	0.17_(110)_	0.865	− 1.09	1.29
Insecure-dismissing	2.99	0.75	2.70	4.81	1.52 _(110)_	0.131	− 0.30	2.28
Insecure-preoccupied	2.70	4.81	1.71	0.76	3.62_(110)_	**0.000**	0.24	0.84
Insecure-disorganized	1.71	0.76	2.11	0.86	1.66_(110)_	0.100	− 0.02	0.27
*Coherence*								
Truth	2.92	0.65	3.08	0.64	− 1.36_(110)_	0.177	− 0.41	0.08
Economy	3.08	0.64	2.75	0.75	− 1.95_(110)_	0.054	− 0.55	0.00
Relation	2.75	0.75	3.02	0.73	− 3.16_(110)_	**0.002**	− 0.65	− 0.15
Manner	3.02	0.73	2.61	0.70	− 0.96_(110)_	0.339	− 0.34	0.12
Overall coherence	2.61	0.70	3.00	0.63	− 1.98_(110)_	0.051	− 0.46	0.00
*Reflective functioning*								
Developmental perspective	2.83	0.74	2.97	0.82	− 0.94_(110)_	0.350	− 0.43	0.15
Theory of mind								
Mother	2.94	0.75	3.25	3.04	− 0.74_(110)_	0.464	− 1.14	0.52
Father	3.25	3.04	2.36	1.01	− 0.59_(107)_	0.555	− 0.47	0.25
Friend	2.36	1.01	2.47	0.90	− 1.15_(109)_	0.253	− 0.41	0.11
Sibling	2.47	0.90	2.85	0.62	− 1.65_(83)_	0.103	− 0.71	0.07
Teacher	2.85	0.62	3.00	0.77	0_(105)_	0.999	− 0.36	0.36
Diversity of feeling								
Self	2.92	0.89	3.13	1.01	− 1.14_(110)_	0.256	− 0.56	0.15
Mother	3.13	1.01	2.94	0.85	− 0.38_(110)_	0.708	− 0.41	0.28
Father	2.94	0.85	3.00	0.96	− 2.27_(108)_	**0.025**	− 0.89	− 0.06
Friend	3.00	0.96	2.31	1.15	1.03_(107)_	0.307	− 0.17	0.54
Sibling	2.31	1.15	2.78	1.03	0.57_(94)_	0.573	− 0.31	0.56
Secure base/safe haven								
Mother	2.28	0.92	2.65	0.86	− 2.22_(110)_	**0.029**	− 0.71	− 0.04
Father	2.10	1.13	2.27	0.88	− 0.89_(108)_	0.374	− 0.56	0.21
*Self-esteem*								
Social competence	3.22	3.02	3.04	0.70	0.43_(110)_	0.668	− 0.64	1.00
School competence	3.04	0.70	3.04	0.61	− 0.90_(110)_	0.369	− 0.33	0.12
Self-regard	3.04	0.61	3.15	0.60	− 2.46_(110)_	**0.016**	− 0.55	− 0.06
*Peer relationship (best friend)*								
Frequency of contact	3.21	0.86	3.05	1.09	0.86_(109)_	0.393	− 0.21	0.53
Quality of contact	3.05	1.09	3.63	4.32	− 0.09_(110)_	0.929	− 1.45	1.32
*Sibling relationship*								
Warmth	2.83	0.79	2.97	0.66	− 0.90_(88)_	0.372	− 0.44	0.17
Hostility	2.97	0.66	1.84	1.00	1.04_(88)_	0.299	− 0.18	0.58
Rivalry	1.84	1.00	1.64	0.81	− 0.54_(88)_	0.588	− 0.43	0.25
*Affect regulation*								
Idealization								
Self	1.53	0.73	1.54	0.73	− 0.13_(110)_	0.898	− 0.29	0.26
Mother	1.54	0.73	1.70	0.82	− 0.57_(110)_	0.573	− 0.36	0.20
Father	1.70	0.82	1.78	0.68	0.99_(109)_	0.325	− 0.17	0.52
Role-reversal								
Mother	1.69	0.70	1.80	1.02	0.95_(110)_	0.342	− 0.13	0.38
Father	1.56	0.69	1.63	0.79	1.66_(109)_	0.099	− 0.05	0.55
Anger								
Mother	1.92	1.07	1.69	0.70	2.07_(110)_	**0.041**	0.02	0.72
Father	1.55	0.79	1.56	0.69	1.15_(109)_	0.254	− 0.14	0.54
Derogation								
Self	1.66	0.85	1.59	0.87	1.79_(110)_	0.076	− 0.03	0.56
Mother	1.39	0.72	1.34	0.72	1.30_(110)_	0.198	− 0.09	0.42
Father	1.41	0.71	1.92	1.07	1.43_(109)_	0.157	− 0.09	0.54
Adaptive response	1.25	0.64	1.55	0.79	−3.30_(110)_	**0.001**	− 0.79	− 0.20
Differentiation of parental representations	3.64	2.98	1.64	1.03	1.15_(110)_	0.253	− 0.35	1.31

The bold values indicate the significant *p*-values

Friends and family interview (FFI)

*n* = 56 in each group

Considering the total sample of the second wave (*N* = 112), higher BES scores showed correlations with higher dismissing (*r* = 0.21, *p* = 0.014), preoccupied (*r* = 0.34, *p* < 0.001) and disorganized (*r* = 0.18, *p* = 0.03) scores. Therefore, dismissing, preoccupied and disorganized patterns were inserted as independent predictors for BES scores in a multiple regression. The final model was strongly significant, explaining 15% of variance of the BES scores, *F* (3,105) = 7.42, *R*^2^ = 0.175 (adjusted* R*^2^ = 0.151), *p* < 0.001, showing a medium effect size, Cohen's *f*^2^ = 0.21. The analysis of coefficients revealed both insecure-dismissing (*β* = 0.22, *p* = 0.015, 95% CI 0.13–1.14) and preoccupied (*β* = 0.32, *p* = 0.001, 95% CI 1.64–5.95) patterns as significant independent predictors for higher BES scores in all girls, the latter as the strongest one.

## Discussion

In the current study, we expanded a previous sample of community adolescents [40] to explore the role of their attachment representations on their levels of binge-eating (BE) tendencies, comparing two groups of girls screened for at risk or not BE behaviors and exploring the role of attachment patterns as predictors of higher BE features.

In the current study, we enlarged a previous sample of community adolescents [40] to explore the role of their attachment representations on their levels of binge-eating (BE) attitudes, both comparing two groups of girls screened at risk or not for BE, and exploring the role of attachment patterns as predictors for more BE.

The initial screening through the BES revealed 7.4% teenagers at risk for binge eating, of which 1.7% revealed severe risk of BE behaviors, and 5.7% resulted at moderate risk (99% girls). If the former fell into the literature range for community adolescents (1–3% [3]), the latter exceeded the peak prevalence reported in literature (4.6% [10]).

Only one boy resulted at risk for BE in the screening, and he refused to be involved in the second wave, allowing us to interpret our results only generalizing them to the female population. According to the literature [11], this result may suggest a possible cultural vulnerability to binge eating in girls from Northern Italy that would deserve to be investigated through national and inter-country epidemiological research within a culturally informed perspective [47].

All the girls screened at risk for binge eating were compared in attachment representations with not-at-risk peers, employing the FFI, an age-adapted attachment interview already used with a part of this sample so far [40], within scarce literature using a narrative approach, more time-consuming but also more informative [36]. Results confirmed that community girls at risk for binge eating appeared more insecure in attachment IWMs than not-at-risk peers, receiving twice as many insecure classifications (52% vs. 25%), in line with clinical adolescents' findings reported in reviews [14, 34–36]. However, unlike clinical teenagers [36], only one girl in the BEG was classified as disorganized, probably due to general lower disorganization rates in community groups with little or no traumatic experiences, compared to clinical and high-risk ones [48, 49].

Albeit insecure, girls in the BEG showed primarily organized attachment IWMs, with a prevalence of preoccupied classifications on dismissing ones, together with higher scores only on insecure-preoccupied pattern’s scale, confirming results of previous research [40]. These findings may support the broader diffusion of attachment entanglement rather than avoidance in individuals showing purging/binge-eating symptoms [50], suggesting that girls with more BE dispositions may be more prone to excessive affective hyperactivation in response to attachment-related stimuli, which may inhibit their exploration of environment and autonomy seeking [24, 26, 27].

In particular, the initial microanalysis on attachment dimensions assessed by FFI's scales allowed to identify the mother as the attachment figure that BEG perceived as less available to provide comfort in case of fear or distress, or rather encourage exploration (i.e., being secure base/safe haven). Our results also suggest that the mother is also the unique source that triggers anger in BEG, as a (defensive) strategy to regulate emotions already observed in clinical ED adults [24]. This dynamic might contribute to their lower adaptive response to upset and negative feelings than NBEG and their ability to recall and tell attachment experiences fluently and coherently (i.e., lower coherence/relation) [15, 45].

Furthermore, in line with the literature findings [33, 35], BEG showed difficulties to recognize and understand personal and others’ mental states (i.e., reflective functioning), and specifically BEG girls struggled to understand the normal co-existence of different feelings, both positive and negative, within the relationship with their fathers. This might suggest a more superficial perception of the paternal relationship that should be further investigated, as community girls that perceive inadequate father’s care were more likely to show BE attitudes in another study [39]. Lastly, in line with the literature, BEG showed lower self-esteem, which may be a source of those negative feelings trigging the “emotional eating” [25]. Also, a consequence of overeating episodes and dieting fails if recurrent, as well as due to difficulties in social relationships might be related to insecure IWMs [25, 36].

However, despite only attachment preoccupation differentiated between girls at risk or not for BE, both insecure dismissing and preoccupied patterns predicted more BE symptoms in the total sample, in line with findings from literature and the previous report [14, 37, 40]. Therefore, girls showing minimization, devaluing and derogation of attachment within significant relationships, and under-activation in response to attachment-related stimuli, may be more vulnerable to display BE behaviors [17, 29, 34–39].

Altogether, these findings support the hypothesis that BE could be a maladaptive strategy to regulate negative emotions (i.e., raised from a lack of self-esteem), when a more adaptive strategy is not available (i.e., seeking emotional support) [25, 34]. For insecure individuals, attachment relationships could be a source of negative feelings in terms of anxiety or anger in case of a preoccupied attachment and unrecognized fear of rejection in dismissing ones, rather than constituting a source of positive feelings and possible help to regulate emotions, eventually favoring the recourse to “emotional eating” (i.e., a tendency to eat in response to overwhelming emotions) [51, 52].

Therefore, these findings suggest the preventive utility to screen BE tendencies in adolescents’ general population to early detect at-risk cases, and then eventually to assess their attachment representations as possibly related to more psychological distress. The use of an age-adapted interview with a dimensional coding system, like the FFI, could help to thoroughly map the adolescent’s domain(s) of vulnerability, to design more targeted interventions [46], and eventually to forecast intervention’s outcomes (e.g., more drop-out in dismissing adolescents and lower efficacy in preoccupied ones) [29].

However, our results need further investigation to be substantiated, due to the several limitations in this study. At first, the screening of BE symptoms was performed only through the BES, that, despite its validity, can be prone to biases related to the exclusive use of questionnaires. Second, a third clinical group with BED could have been helpful both to understand the role of attachment disorganization and to better examine similarities and differences in BE between community and clinical adolescents. Third, the higher prevalence of girls in the 1st wave (67%) reduced the probability to find boys at risk for BE. Also, the inclusion of boys in our sample for the 2nd wave was not possible because the only boys at risk for BE refused to participate in this phase of the study; this issue permits us to interpret our results only generalizing them to the female population. Further, participants came from the North-west part of Italy, limiting their representativeness to the whole Italian population, and no other factors related to BE were explored, such as emotion regulation strategies [25]. Lastly, causal connections analyzed through prediction models could not be fully validated within a correlational design. Therefore, future studies should involve larger mixed-gender clinical and community groups—at risk or not for BE—from different parts of Italy, with screening through a multi-method approach and measuring further vulnerability factors, possibly employing a longitudinal design.

### What is already known on this subject?

Insecure attachment is a risk factors for BED in clinical samples and general population, but only one previous study used an in-depth attachment interview to investigate the role of insecure patterns (preoccupied, dismissing and disorganized) as risk factors for more binge-eating features in non-clinical adolescents.

### What does this study add?

For the first time, this study compared non-clinical girls at risk or not for binge eating considering multiple dimensions of attachment (e.g., social competence, reflective functioning, affective regulation strategies) and not only broader classifications. Such dimensional assessment could help map dimensions of vulnerability and resilience in teenagers, plan prevention and intervention on binge-eating symptoms in community adolescents.

## Data Availability

Data available on request.

## Code availability

Custom code.

## References

[1] American Psychiatric Association (2013). Diagnostic and statistical manual of mental disorders.

[2] Galmiche M, Déchelotte P, Lambert G, Tavolacci MP (2019). Prevalence of eating disorders over the 2000–2018 period: a systematic literature review. Am J Clin Nutr.

[3] Bohon C (2019). Binge eating disorder in children and adolescents. Child Adolesc Psychiatr Clin.

[4] Brownley KA, Berkman ND, Peat CM (2016). Binge-eating disorder in adults: a systematic review and meta-analysis. Ann Intern Med.

[5] Murray SB, Griffiths S, Nagata JM (2018). Community-based eating disorder research in males: a call to action. J Adolesc Health.

[6] American Psychiatric Association (APA) (2000) Diagnostic and statistical manual of mental disorders (4th edn., text rev.). APA, Washington, DC

[7] Coelho JS, Kumar A, Kilvert M, Kunkel L, Lam PY (2015). Male youth with eating disorders: clinical and medical characteristics of a sample of inpatients. Eat Disord.

[8] Mitchison D, Mond J (2015). Epidemiology of eating disorders, eating disordered behaviour, and body image disturbance in males: a narrative review. J Eat Disord.

[9] Støving RK, Andries A, Brixen K, Bilenberg N, Hørder K (2011). Gender differences in outcome of eating disorders: a retrospective cohort study. Psychiatry Res.

[10] Marzilli E, Cerniglia L, Cimino S (2018). A narrative review of binge eating disorder in adolescence: prevalence, impact, and psychological treatment strategies. Adolesc HealthMedTher.

[11] Pace CS, Muzi S (2019). Binge-eating symptoms, emotional-behavioral problems and gender differences among adolescents: a brief report. Mediterr J Clin Psychol.

[12] Allen KL, Byrne SM, Oddy WH, Crosby RD (2013). DSM–IV–TR and DSM-5 eating disorders in adolescents: prevalence, stability, and psychosocial correlates in a population-based sample of male and female adolescents. J Abnorm Psychol.

[13] Goldschmidt AB, Wall MM, Loth KA, Bucchianeri MM, Neumark-Sztainer D (2014). The course of binge eating from adolescence to young adulthood. Health Psychol.

[14] Faber A, Dubé L, Knäuper B (2018). Attachment and eating: a meta-analytic review of the relevance of attachment for unhealthy and healthy eating behaviors in the general population. Appetite.

[15] Tasca GA, Balfour L (2014). Attachment and eating disorders: a review of current research. Int J Eat Disord.

[16] Bowlby J (1982). Attachment and loss: retrospect and prospect. Am J Orthopsychiatry.

[17] George C, Kaplan N, Main M (1985). Theadultattachmentinterview.

[18] Main M, Goldwyn R, Hesse E (2008). Adultattachmentscoringandclassificationsystem.

[19] Kobak R (1999) The emotional dynamics of disruptions in attachment relationships: implications for theory, research, and clinical intervention. ISO 690

[20] Benzi IMA, Di Pierro R, De Carli P, Cristea IA, Cipresso P (2020). All the faces of research on borderline personality pathology: drawing future trajectories through a network and cluster analysis of the literature. J Evid Based Psychother.

[21] Brennan KA, Clark CL, Shaver PR (1998) Self-report measurement of adult attachment: an integrative overview.

[22] Tanofsky-Kraff M, Wilfley DE, Young JF, Mufson L, Yanovski SZ, Glasofer DR, Salaita CG (2007). Preventing excessive weight gain in adolescents: interpersonal psychotherapy for binge eating. Obesity.

[23] Rieger E, Van Buren DJ, Bishop M, Tanofsky-Kraff M, Welch R, Wilfley DE (2010). An eating disorder-specific model of interpersonal psychotherapy (IPT-ED): causal pathways and treatment implications. Clin Psychol Rev.

[24] Caglar-Nazali HP, Corfield F, Cardi V, Ambwani S, Leppanen J, Olabintan O, Deriziotis S, Hadjimichalis A, Scognamiglio P, Eshkevari E, Micali N, Treasure J (2014). A systematic review and meta-analysis of ‘Systems for Social Processes’ in eating disorders. Neurosci Biobehav Rev.

[25] Cortés-García L, Takkouche B, Seoane G, Senra C (2019). Mediators linking insecure attachment to eating symptoms: a systematic review and meta-analysis. PLoS ONE.

[26] Kuipers SG, Bekker HJM (2012). Attachment, mentalization and eating disorders: a review of studies using the adult attachment interview. Curr Psychiatry Rev.

[27] O'Kearney R (1996). Attachment disruption in anorexia nervosa and bulimia nervosa: a review of theory and empirical research. Intl J of Eat Disord.

[28] O'Shaughnessy R, Dallos R (2009). Attachment research and eating disorders: a review of the literature. Clin Child Psychol Psychiatry.

[29] Tasca GA (2019). Attachment and eating disorders: a research update. Curr Opin Psychol.

[30] Ward A, Ramsay R, Treasure J (2000). Attachment research in eating disorders. Br J Clin Psychol.

[31] Zachrisson HD, Skårderud F (2010). Feelingsofinsecurity:reviewofattachmentandeatingdisorders. Eur Eat Disord Rev.

[32] Barone L, Guiducci V (2009). Mental representations of attachment in eating disorders: a pilot study using the adult attachment interview. Attach Hum Dev.

[33] Maxwell H, Tasca GA, Grenon R, Faye M, Ritchie K, Bissada H, Balfour L (2018). Change in attachment dimensions in women with binge-eating disorder following group psychodynamic interpersonal psychotherapy. Psychother Res.

[34] Gander M, Sevecke K, Buchheim A (2015). Eating disorders in adolescence: attachment issues from a developmental perspective. Front Psychol.

[35] Jewell T, Collyer H, Gardner T, Tchanturia K, Simic M, Fonagy P, Eisler I (2016). Attachmentandmentalization and theirassociationwithchildandadolescenteatingpathology:asystematicreview. Int J Eat Disord..

[36] Salcuni S, Parolin L, Colli A (2017). Attachment research and eating disorder: a measurement perspective-literaturereview. PFP.

[37] Boone L (2013). Are attachment styles differentially related to interpersonal perfectionism and binge eating symptoms?. Pers Individ Differ.

[38] Han S, Pistole MC (2014). College student binge eating: insecure attachment and emotion regulation. J Coll Stud Dev.

[39] Pace U, Cacioppo M, Schimmenti A (2012). The moderating role of father’s care on the onset of binge eating symptoms among female late adolescents with insecure attachment. Child Psychiatry Hum Dev.

[40] Pace CS, Muzi S, Calugi S, Dalle Grave R (2020). Attachment representations and alexithymia in community adolescents with binge-eating attitudes. Eat Weight Disord.

[41] Benzi IMA, Fontana A, Di Pierro R, Perugini M, Cipresso P, Madeddu F, Clarkin JF, Preti E (2021). Assessment of personality functioning in adolescence: development of the adolescent personality structure questionnaire. Assessment.

[42] Benzi I, Sarno I, Di Pierro R (2018) Maladaptive personality functioning and non-suicidal self-injury in adolescence. Clin Neuropsychiatry 15(4)

[43] Gormally J, Black S, Daston S, Rardin D (1982). The assessment of binge eating severity among obese persons. Addict Behav.

[44] Di Bernardo M, Barciulli E, Ricca V, Mannucci E, Moretti S, Cabras PL, Rotella CM (1998). Validazione della versione Italiana della binge eating scale in pazienti obesi. Minerva Psichiatr.

[45] Steele H, Steele M, Kriss A (2009) FFI scoring system. New York, NY: Unpublished Manuscript, Center for Attachment Research, New School for Social Research

[46] Pace CS, Muzi S, Steele H (2020). Adolescents’ attachment: content and discriminant validity of the friends and family interview. J Child Fam Stud.

[47] Miller MN, Pumariega AJ (2001). Culture and eating disorders: a historical and cross-cultural review. Psychiatry.

[48] Bakermans-Kranenburg MJ, van IJzendoorn MH (2009). The first 10,000 adult attachment interviews: distributions of adult attachment representations in clinical and non-clinical groups. Attach Hum Dev.

[49] Cassibba R, Sette G, Bakermans-Kranenburg MJ, Van Ijzendoorn MH (2013). Attachment the Italian way. Eur Psychol.

[50] Dias P, Soares I, Klein J, Cunha JP, Roisman GI (2011). Autonomic correlates of attachment insecurity in a sample of women with eating disorders. Attach Hum Dev.

[51] Han S, Kahn JH (2017). Attachment, emotion regulation difficulties, and disordered eating among college women and men. J Couns Psychol.

[52] Santona A, De Cesare P, Tognasso G, De Franceschi M, Sciandra A (2019). The mediating role of romantic attachment in the relationship between attachment to parents and aggression. Front Psychol.

